# Analysis of cross neutralizing activity of antibodies from sera of severe fever with thrombocytopenia syndrome patients to deal with different genotype strains

**DOI:** 10.3389/fmicb.2022.1020545

**Published:** 2022-10-20

**Authors:** Jiaxin Xu, Yuanni Liu, Fan Zhang, Xin Wang, Weijin Huang, Yulong Wu, Boqing Li, Jiapeng Zhuang, Yixing Bing, Youchun Wang, Yuanyuan Qiao

**Affiliations:** ^1^Department of Pathogenic Biology, Binzhou Medical University, Yantai, China; ^2^Department of Infectious Diseases and Clinical Laboratory, Yantai Qishan Hospital, Yantai, China; ^3^Key Laboratory for Quality Research and Evaluation of Biological Products, National Medical Products Administration, Beijing, China

**Keywords:** severe fever with thrombocytopenia syndrome bunyavirus, humoral immunity, neutralizing antibody, glycoprotein, phylogenetic tree

## Abstract

**Background:**

Severe fever with thrombocytopenia syndrome bunyavirus (SFTSV) is a tick-borne virus that causes severe communicable fever with thrombocytopenia syndrome (SFTS) with an average case fatality rate of 10%. In the study, we aimed to identify the cross-neutralizing antibody (nAb) against different genotype strains from sera of SFTSV infected patients.

**Methods:**

Firstly the genotype of SFTSV was identified by constructing a phylogenetic tree based on the M segments epidemic in the Jiaodong area of Shandong province, then different sera of subjects cross reactive with recombinant Gn (rGn-Fc) or recombinant Gc (rGc-Fc) of 0921 strain were examined. The levels of polyclonal nAbs from sera of 25 convalescents were measured by a pseudovirus-based neutralizing experiment.

**Results:**

We found local endemic strains were mainly C2 and C3 isolates of SFTSV. 14 of 15 sera from donors reacted with 0921 rGn-Fc, and 9 of 15 sera from donors reacted with 0921 rGc-Fc. Cross nAbs were produced by 10 of 25 sera from donors during the period of 2019–2021. Among these, five nAbs (A2, A4, A5, L9, and L10) neutralized the pseudoviruses of HB29, Gangwon, HN13, HN20, SPL030A, and SD4 strains.

**Conclusion:**

Our data suggested that epidemic strains showed relatively stable heredity. Some blood sources from patients produced cross nAbs that could neutralize all of the strains examined. These findings highlight the important role played by humoral immunity in combatting SFTSV.

## Introduction

Severe fever with thrombocytopenia syndrome (SFTS) is primarily characterized by fever, thrombocytopenia, and leukocytopenia. It was first reported in China by Yu et al. (2011) and has an average case fatality rate of 10% (Yu et al., 2011; Li, 2013). It is a communicable disease transmitted by a tick-borne SFTS bunyavirus (SFTSV; Li, 2013; Yoo et al., 2016). In eastern China, people are generally infected with the virus in two peak seasons. The number of SFTSV cases rises generally in April and in the end of October. The most susceptible group of people are farmers and outdoor workers (Wang et al., 2015).

**Figure 1:**
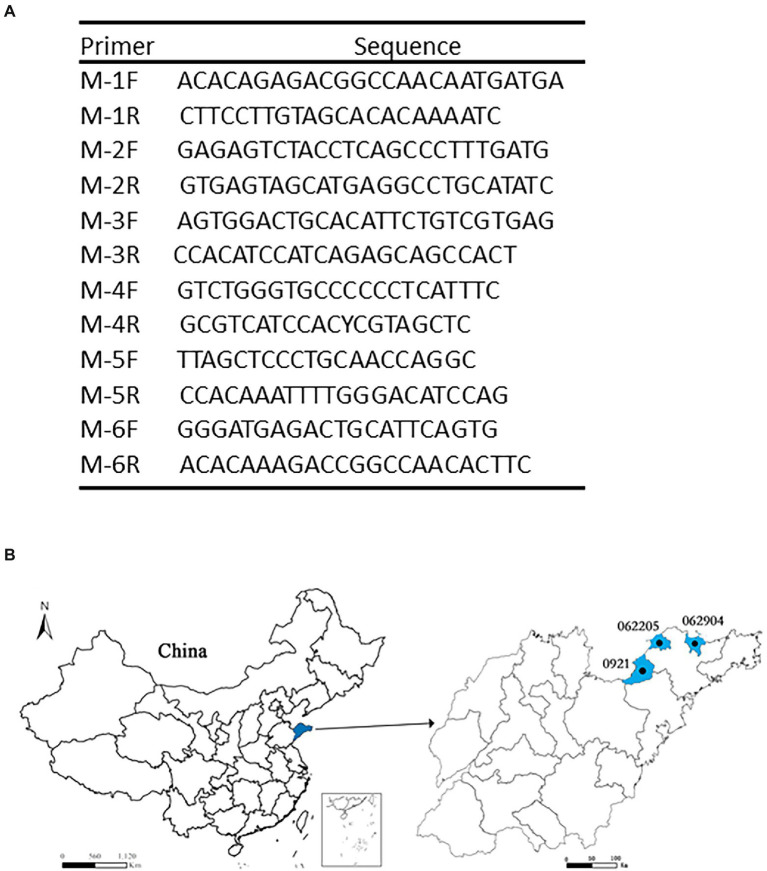
The geography distribution of SFTSV studied. **(A)** The six primers pairs were showed. From M-1F, M-1R to M-6F, M-6R. **(B)** The geography distribution of SFTSV. The blue part showed the Jiaodong area in Shandong province on the left, and the blue on the right is the distribution of different clades (0921, 062205, and 062904).

In China, the majority of SFTS cases have been reported in Henan, Shandong, Anhui, and Hubei Provinces, accounting for over 90% of the total cases. So far, C1, C2, C3, C4, and similar genotype strains are mainly epidemic in China, and J1, J2, and J3 are mainly epidemic in Japanese and Korean populations (Shi et al., 2017; Wu et al., 2021). Due to the special geographical location of the Jiaodong area of Shandong Province, the infection rates and fatality rates are considerably high in the region (Figure 1B). However, the clade of the local epidemic strains is yet unknown. Therefore, identification of genotypes of SFTSV contributing to the local prevalence is urgently required in order to prevent and control the spread.

SFTSV is a negative single-stranded segmented RNA virus, which has a genome consisting of S, M, and L segments (Niu et al., 2013). Gn/Gc encoded by the M segment of SFTSV are the glycoprotein on the surface of SFTSV responsible for attachment and membrane fusion, which is required for host cell entry (Halldorsson et al., 2016; Wu et al., 2017). Gn/Gc as an important pathogenic factor had ideal regions recognized by specific broad and potent nAbs (Wang et al., 2020). Several membrane receptors recognized by Gn/Gc were previously reported to involve in the SFTSV entry into cells. DC-SIGN was considered as a responsible receptor for SFTSV (Garcia-Vallejo et al., 2013; Hofmann et al., 2013). Moreover, non-muscle myosin heavy chain IIA binding to Gn during viral infection, was reported the disruption function led to thrombocytopenia (Sun et al., 2014). To explore the binding region of potential monoclonal nAb from patient, we examined the binding ability of different sera from donors with recombinant Gn (rGn-Fc) and recombinant Gc (rGc-Fc) exposed on the surface of SFTSV.

To date, no immunoprophylaxis and therapies are known for SFTS. In a study by Fang et al., they reported that the titer of neutralizing antibody (nAb) decreases over time during a 4-year period (Huang et al., 2016). Yoo et al. reported nAb in one healthy resident (Jeju-H21) persisted for the entire study period of 3 years (Yoo et al., 2021). Cao et al. reported nAb from sera of subject X was firstly examined after he was admitted to hospital for 9 days (Cao et al., 2022). Two monoclonal nAbs, named MAb 4-5 and Ab10, neutralized different isolates of SFTSV (Guo et al., 2013; Kim et al., 2019). In another study by Wu et al. on a humanized mouse model, they found a single-domain antibody SNB02 inhibited SFTSV infection and prevented thrombocytopenia. They proposed SNB02 as a potential therapeutic agent for SFTS (Wu et al., 2020). Primarily all known strains of SFTSV can be divided into two lineages that is C lineage and J lineage (Won et al., 2019). Unfortunately, there are no clear reports on any cross nAb toward different genotype strains of SFTSV. In order to evaluate the ability of humoral immunity against SFTSV, it is necessary to detect the level of nAbs from sera against different genotypes. In the future, this can help in the development and designing of single or combination of monoclonal nAb drugs and vaccines. This study examined neutralizing ability of IgG from convalescence subjects toward different pseudoviruses of five genotypes by pseudovirus-based neutralization assay.

In this study, sera were collected from 43 patients infected with SFTSV between 2019 and 2021. The genotype of M segment of SFTSV was identified from three acute patients infected with SFTSV in 2021. 15 sera from donors were used to examine the binding reactivity with rGn-Fc/rGc-Fc of 0921 strain. Moreover, the cross-neutralizing ability of antibodies toward six different strains of SFTSV was assessed from sera of 25 patients.

## Materials and methods

### Study sample and pseudoviruses

Forty-three sera and blood specimens were collected during the year of 2019 and 2021, among of these, No. 1-9, Q1-44 were specimens collected from convalescent phase, and 0921, 062904, and 062205 were samples collected from admission day. Statistical data of gender, age, virus loads, alanine aminotransferase, troponin level, and platelet of donors were obtained from Yantai Qishan Hospital. Pseudoviruses HB29 (C3 genotype), Gangwon (C2 genotype), HN13 (C4 genotype), HN20 (J genotype), SPL030A (J genotype), and SD4 (C1 genotype) were packaged by Key Laboratory for quality research and evaluation of Biological Products, National Medical Products Administration.

### Polyclonal antibody IgG purified from sera

Polyclonal antibody IgGs were purified from sera specimens to avoid the interruption of complements and drugs in sera. Briefly, sera from convalescent patients were collected and incubated at 56°C for 30 min followed by centrifugation. After filtration IgGs were isolated by affinity purification using protein A beads which were combined with IgG_1_, IgG_2_, and IgG_4_ from Human (Sino Biological). The purity and molecular weight of the antibodies were confirmed by SDS-PAGE.

### M segments of SFTSV from different acute stage patients

Sera specimens were collected from three patients in the Jiaodong area of Shandong Province. RNA extraction was from the patient’s serum specimens, followed by RT-PCR, then six primer pairs were used for gene amplification using Fast Pfu polymerase (Figure 1A). Amplified fragments were sent to the company for sequencing. DNA sequences were ligated to form M segments of the genome of SFTSV.

### Phylogenetic tree constructed using the sequence of M segment

The amine acids sequences of M segments were searched by BLAST in the GeneBank database, followed by the selection of highly similar sequences with M segments of SFTSV, then saved as fasta format. The M amine acids sequences were aligned by clustal W, exported alignment and saved as mega format. The phylogenetic tree was constructed using Neighbor-Joining algorithm choosing bootstrap method by Mega 11 (Mega 6.0 software updated version; Tamura et al., 2013).

### ELISA

Recombinant proteins Gn (rGn-Fc) and recombinant Gc (rGc-Fc) of 0921 strain was expressed and then purified from 293T cells transiently transfected with pHL vector linked with SFTSV 0921 Gn/Gc nucleic acid sequence and human IgG1 Fc fragment. Briefly, rGn-Fc/rGc-Fc was coated on 96 plate with 1 μg/ml for 50 μl, then blocked with 5% nonfat milk for 2 h, different sera from SFTS donors diluted with 1:500 for 50 μl were added into plate for 1 h, and washed for four times with 1% PBST, then HRP-anti-human-Fab (Jackson) was added into each well, each well received 50 μl of 3, 3′, 5, 5′-tetramethylbenzidine (TMB, Sigma) substrate solution for 1 h. The color reaction was terminated by 2 M H_2_SO_4_. The absorbance of each well was measured at 450 nm using a microplate spectrophotometer (Molecular Devices).

### Neutralization assays

Neutralization of IgGs purified from sera of patients was measured using huh7 cells infected in a single-round invasion with SFTSV pseudovirus (Chen et al., 2020). SFTSV pseudoviruses HB29, Gangwon, HN13, HN20, SPL030A, and SD4 were generated by co-transfecting 293T cells with VSVΔG-Fluc and SFTSV envelope plasmids. Post 36–48 h of transfection, the supernatant containing pseudoviruses was harvested, centrifuged, and the titer of pseudovirus was determined. The sample was frozen at −80°C until the next use. In the neutralization assay, three-fold serial dilution of 50 μl of 50 μg/ml IgG was incubated with 50 μl of pseudoviruses in a 96-well cell culture plate at 37°C for 1 h before the addition of huh7 cells. After 36–48 h of incubation, the cells were lysed, and the viral infectivity was quantified by measuring luciferase activity with a chemiluminescence detector (Tecan). The 50% inhibitory concentration (IC_50_) was calculated as the antibody concentration that reduced the infection of pseudoviruses by 50%.

### Statistical analysis

GraphPad Prism 6 was used for statistical analyses of neutralizing activity of IgG and binding activity of rGn-Fc/rGc-Fc.

## Results

### Case clinical features and surveillance

Sera and blood specimens were collected from 43 SFTS patients. 28 of 43 cases clinical features were collected and analyzed. Among of these 28 cases, the male to female ratio is 17/11, and mean age is 63.8 years old. All of the patients showed thrombocytopenia, ranging from 12 to 99 (E+09/L) with a median value of 44 (E+09/L) (Table 1), and a more than 10-fold severe decline of blood platelets in patient (No. 6). All of the patients were reported with alanine transaminase elevation, ranging from 40.5–583.9 U/L (Table 1), with a median value of 128.2 U/L, and the highest ALT was 14 fold above the normal value. Elevated troponin levels were reported in 27 of 28 patients ranging between 6.7–3,760 pg./ml (Table 1), with a median value of 93.3 pg./ml. The highest troponin was 241-fold above the normal value (No. 0921, 71 years old). On admission of the patients, the virus load was between 51 and 10^7^ (copies/ml; Table 1), and the median value was 2.72E+03 (copies/ml). Overall, SFTS patients showed thrombocytopenia, liver damage, and cardiac damage, along with other severe complications.

**Table 1:** The characteristics of convalescent of SFTS.

No. of patient	No. of antibody/genotype	Gender (M/F)	Age	The first test of copies of virus	The last test of virus load at discharge	The highest ALT	Discharged ALT	The worst troponin	Discharged troponin	The lowest PLT	Discharged PLT	Severity (F/N)
1	A1	M	86	6.06E+03	TND	420	44.06	243.2	80.07	66	414	N
2	A2	M	71	2.44E+03	TND	72.1	28	22.6	13.1	55	325	N
3	A3	M	58	1.87E+03	TND	191	64.58	81.4	12.3	49	154	N
4	A4	M	81	3.17E+03	5.43	130	28.85	535.6	19.7	40	466	N
5	A5	M	57	2.72E+03	TND	223.1	57.5	69.2	21.3	41	328	N
6	A6	M	67	2.17E+04	TND	89.69	20.67	1931.6	2.8	12	226	N
7	A7	M	73	2.02E+04	TND	212	37.81	41.6	9.1	56	242	N
8	A8	F	65	2.36E+02	TND	99.27	24.48	98	17.1	66	341	N
9	A9	M	74	3.09E+03	TND	50.1	35.83	140.3	32.1	74	325	N
Q1	L1	M	64	6.83E+04	TND	168.9	57.9	248.1	26	35	164	N
Q2	L2	F	52	8.32E+02	TND	65.7	28.5	39.7	18.3	56	195	N
Q8	L8	M	68	2.51E+02	TND	126.4	49	21.1	14.8	31	218	N
Q9	L9	F	62	7.47E+02	TND	143.1	17.4	511.9	10	26	240	N
Q10	L10	F	42	3.64E+02	TND	87	19.9	23.1	8.2	74	302	N
Q11	L11	M	69	1.40E+03	TND	229.6	47.4	59.2	18.9	33	139	N
Q14	L14	F	52	8.85E+03	TND	45.4	13.4	574.9	8	14	151	N
Q16	L16	M	57	5.10E+01	TND	128.2	33.5	41.8	1	99	417	N
Q18	L18	M	42	2.85E+02	TND	270.5	27.1	34.1	1.2	44	260	N
Q19	L19	M	59	1.75E+03	TND	79.3	48	6.7	5.1	44	189	N
Q22	L22	M	58	4.58E+02	TND	402.2	69.7	48.1	1.4	60	276	N
Q23	L23	M	84	3.02E+03	TND	50.8	22.4	93.3	36.6	26	118	N
Q29	L29	F	66	2.85E+03	TND	262.6	21.6	938.9	4.6	98	337	N
Q30	L30	F	56	1.12E+03	TND	40.5	28.7	172.3	19	68	241	N
Q33	L33	F	41	2.85E+03	TND	56.3	56.1	73.9	40.6	53	206	N
Q44	L44	F	66	7.15E+03	TND	196.6	51.7	126	51.2	56	260	N
0921	C2	M	71	E7	ND	583.9	ND	3,760	ND	30	99	F
062904	C3	F	65	8.28E+04	2.83E+05	216	78.3	1,315	ND	33	ND	F
062205	C2	F	82	3.57E+04	ND	53.2	ND	554	ND	22	ND	F

Target not determined (TND) represented virus load below the sensitivity by fluorescence probe PCR. Not determined (ND) represented the part was not tested. E+09: ×109. Gender (M/F), M, male, F, female. The normal range of ALT is 7–40 U/L. The normal range of troponin: 0–15.6 pg/ml. The normal range of PLT: 125–350 E+09/L. Virus load (copies/ml), ALT (U/L), troponin (pg/ml), PLT ((E+09)/L). Severity (F/N): F, fatal, N, nonfatal.

### The genotype of strains epidemic in the Jiaodong area

**Figure 2:**
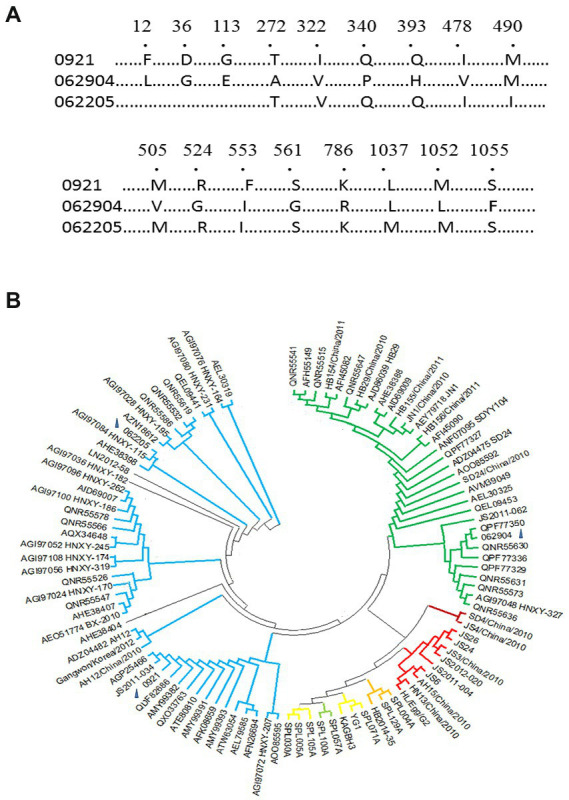
Clades and phylogenetic tree of M segments of three novel isolates. **(A)** M segments of isolates (0921, 062205, and 062904), Number showed the different amino acids among of them (1,073 amino acids of M segment). **(B)** The M phylogenetic tree of the three isolates with 99 different isolates was aligned using Mega 11 (Mega 6.0 software updated version). According to the M phylogenetic tree, 0921 and 062205 belonged to the C2 clade. 062904 belonged to the C3 clade. Clades C1-C4 and J are represented with different colors: Green-C3 clade, Crimson-C1 clade, Red-C4 clade, Blue-C2 clade, Yellow-J clade. The blue triangle marked the loci of 0921, 062904, and 062205 on the phylogenetic tree.

To define the clades of SFTSV infected, we identified genotype of three strains (0921, 062904, and 062205) based on M segments obtained by RT-PCR from sera of patients in the acute stage in Jiaodong area (Figure 2A). The accession numbers of M segments of 0921, 062904, and 062205 are OM162020, OP485773, and OP485774 in BanKIt database. BLAST alignment and phylogenetic analyses of the M segments of SFTSV strains were done. 0921 shared a high amino acid identity (100%) with previously isolated SFTSV strains of SDLZtick12/2010, and 062904 shared a 100% identity with the 17-China_Henan-72 strain. In addition, 062205 has a 99.76% identity with 15-China_Henan-380 strain. In the three strains, 0921 shared the difference of 15 amino acids of M segment with 062904, among of them, seven amino acids were located on Gn (1–452) of SFTSV, and one amino acid (K786R) was located on Gc (563–996). Thus, based on the previously reported references, 0921 and 062205 belonged to the C2 clade, and 062904 belonged to the C3 clade (Figure 2B; Shi et al., 2017; Wu et al., 2021). These results showed that the strains in the Jiaodong area have a high identity with Henan Province and are mainly from C2 and C3 genotypes.

**Figure 3:**
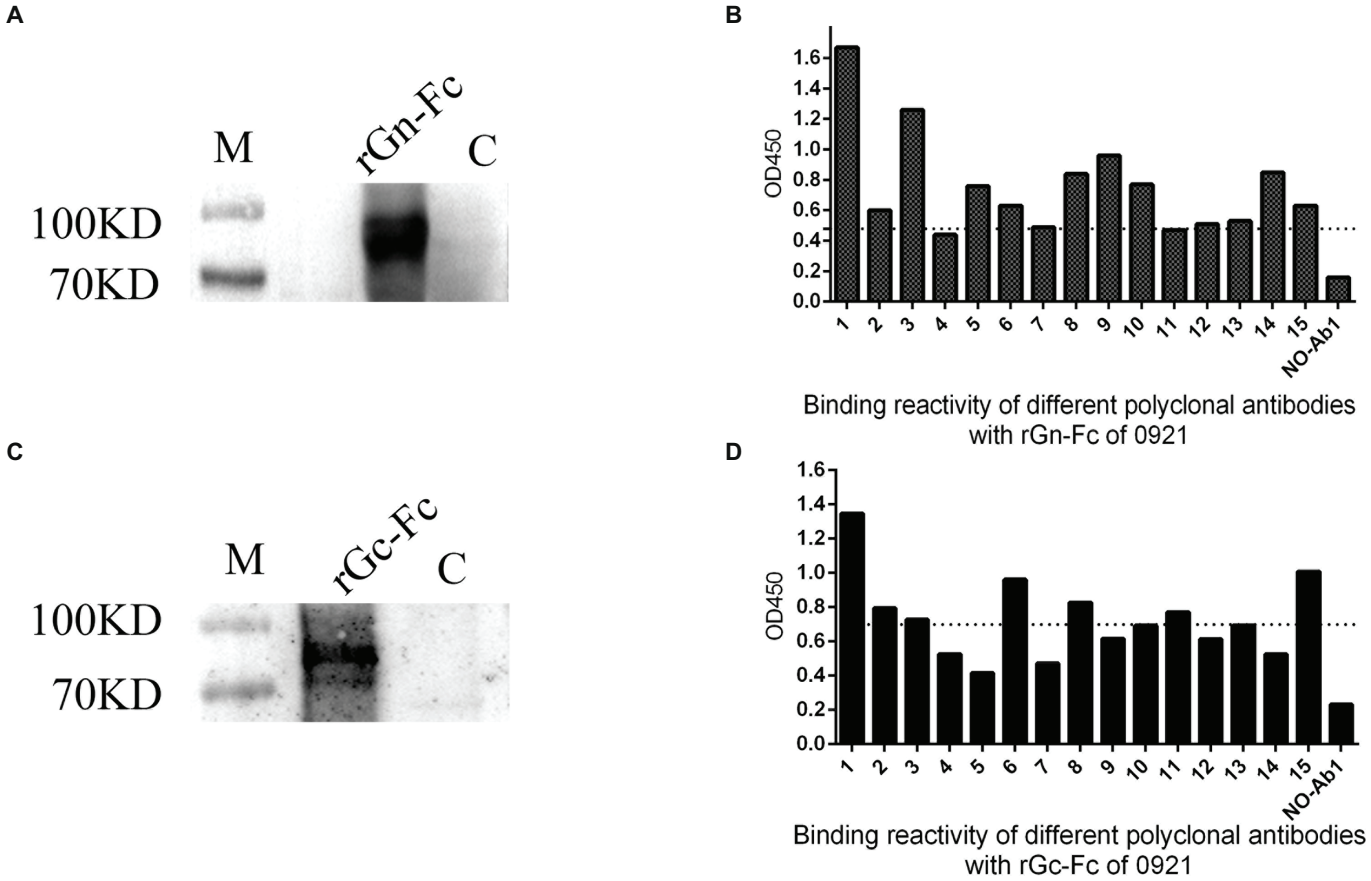
Cross reactivity of rGn-Fc/rGc-Fc of 0921 strain with different sera from SFTS donors. **(A)** rGn-Fc of 0921 strain by Western blot. The molecular weight of rGn-Fc is about 90KD. **(B)** Sera from SFTS donors reacted with rGn-Fc by ELISA. 15 sera were collected from donors in 2021. 14 of 15 sera reacted with rGn-Fc. The dotted line showed cut-off value. **(C)** rGc-Fc of 0921 strain by Western blot. The molecular weight of rGc-Fc is about 90KD. **(D)** Sera from SFTS donors reacted with rGc-Fc by ELISA. 9 of 15 sera reacted with rGc-Fc. The dotted line showed cut-off value.

To explore variability of SFTSV, we expressed and purified rGn-Fc/rGc-Fc of 0921 strain (Figures 3A,C), then examined the binding reactivity of different sera with 0921 Gn/Gc which was exposed on the surface of SFTSV, we found 14 of 15 sera of donors infected showed the obviously reactivity with 0921 rGn-Fc (cut-off value, 0.47; Figure 3B), and 9 of 15 sera of donors infected showed the obviously reactivity with 0921 rGc-Fc (cut-off value, 0.69; Figure 3D), and the cut-off value was determined by calculating three-fold of the mean OD450 of a negative control.

### Some patients produced cross neutralizing antibodies

**Figure 4:**
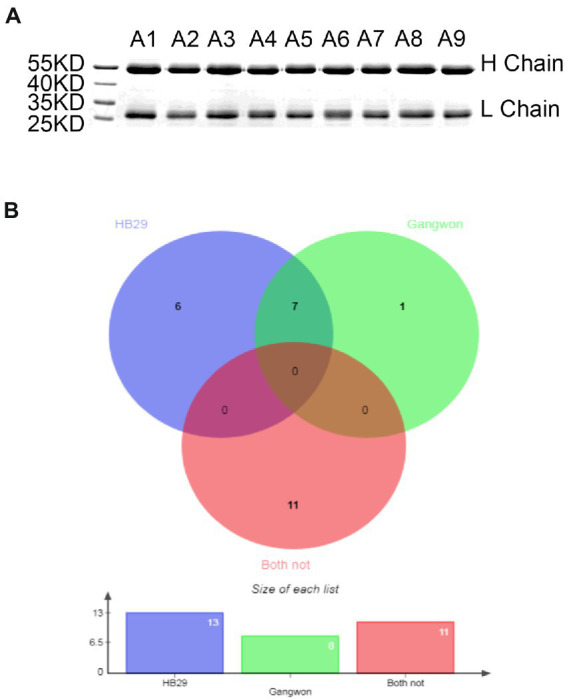
The neutralizing activity of IgG from 25 different patients against pseudovirus of HB29 and Gangwon. **(A)** IgG purified from sera of convalescent by SDS-PAGE. The molecular weight of the heavy chain is 50 KD, and the Light chain is over 25 KD. The purity is over 95%. **(B)** Venn diagram shows 25 nAbs that neutralized pseudovirus HB29 and Gangwon strain analyzed using online software. Violet blue highlights 13 nAbs that neutralized HB29 pseudovirus, green highlights eight nAbs that neutralized Gangwon pseudovirus, and red highlights 11 nAbs that neutralized neither HB29 pseudovirus nor Gangwon pseudovirus. There are seven nAbs that neutralized both HB29 and Gangwon pseudovirus.

To assess the ability of the humoral immune system of patients with SFTSV, we examined the neutralization of antibodies from different patients. Polyclonal antibody IgGs were isolated from serum identified using protein A bead, to avoid the disturbance of complements and medicines (Figure 4A). During the period 2019–2021, 25 polyclonal antibodies were purified from 25 convalescent patients. Firstly, we examined the antibodies toward HB29 and Gangwon strains. We found that 14 of 25 patients produced nAbs. Among them, A1, A9, L8, L14, L23, and L33 neutralized HB29 strain but not Gangwon strain, whereas, A2, A4, A5, L9, L10, L18, L9 neutralized HB29 strain and Gangwon strain, and L22 neutralized Gangwon strain but not HB29 strain (Figure 4B). Then we examined A1, A2, A4, A5, A9, L9, L10, L14, L18, L19, L22, L23, and L33 toward the four strains (HN13, HN20, SPL030A, and SD4) and found neutralizing antibodies from the sera of 5 patients (2, 4, 5, Q9, Q10) could neutralize the four strains. L19 neutralized HN20, SPL030A, and SD4 strains. L14 neutralized HN20 and SD4 strains. L18 neutralized HN20 and SPL030A strains. L23 and L33 neutralized HN20 strain (Table 2). In general, when the six strains of SFTSV were examined, nAbs A2, A4, A5, L9, and L10 could successfully neutralize all of the six strains of five genotypes, L19 neutralized five strains, L18 neutralized four strains, L14 neutralized three strains, and L23 and L33 neutralized two strains.

**Table 2:** Thirteen of 25 nAbs neutralized pseudovirus of HB29, Gangwon, HN13, HN20, SPL030A, and SD4 strains.

No. of antibody	IC50 (μg/ml)					
	HN13	HN20	SPL030A	SD4	HB29	Gangwon
A1	>50	>50	>50	>50	4.46	>50
A2	5.7	3.01	6.77	3.18	6.88	3.06
A4	4.93	2.41	1.53	4.13	6.33	1.90
A5	1.83	3.05	3.32	5.58	24.08	21.05
A9	>50	>50	>50	>50	0.73	>50
L9	7.9	2.19	5.16	3.66	0.46	0.39
L10	12.93	8.67	30.22	11.42	8.12	9.73
L14	>50	15.51	>50	8.01	33.45	>50
L18	>50	0.86	2.33	>50	22.04	3.25
L19	>50	0.37	50	50	0.33	0.12
L22	>50	>50	>50	>50	>50	1.68
L23	>50	17.2	>50	>50	12.25	>50
L33	>50	1.29	>50	>50	1.46	>50

## Discussion

The SFTSV infection is prevalent in Central and Northeast China, Korea, Japan, and Vietnam (Yu et al., 2011; Kim et al., 2013; Takahashi et al., 2014; Maslow et al., 2019; Tran et al., 2019). In China, it has been reported in Henan, Anhui, Hubei, Jiangsu, Shandong, and Liaoning Province (Zhan et al., 2017; Wu et al., 2021). In recent years, there had been a dramatic increase in the outbreaks and mortality rate of SFTS. In eastern China, reportedly, the data showed that SFTSV infected more males than females and people aged ≥60 years more than 40–59 (Antonelli et al., 2021), which was consistent with our findings in Jiaodong area of Shandong province. The virus load of patients on admission was 10^2^–10^7^, and the viruses were eliminated when SFTS patients were fully recovered. During the period 2019–2021, the main index including blood platelet, ALT, and troponin of SFTS patients was examined from the blood specimens, therefore, they showed the signs and symptoms of thrombocytopenia, hepatic injury, cardiac damage. In addition, the signs and symptoms of fever, gastrointestinal symptoms, leukocytopenia, and even nerve system injury already reported in SFTS patients. However, post-discharge, the anomaly index of inpatients gradually returned to normal levels.

There are C lineage and J lineage clades of SFTSV in China (Wu et al., 2021). To ascertain the strains causing an epidemic in the Jiaodong area of Shandong Province, we attempted to analyze the genotype of SFTSV strains. We found C2 and C3 were the main epidemic strains here, where 0921 shared a 100% identity with SDLZtick12/2010 isolated from *Haemaphysalis longicornis* in 2010, and rGn-Fc/rGc-Fc of 0921 cross reactive with different sera from subjects. Although 0921 was from the serum of an acute patient infected with SFTSV in 2021, it suggested that the glycoprotein encoding by M segments was stable, despite the fact that SFTSV is a segmented RNA virus. In addition, we found polyclonal antibodies mainly target Gn of SFTSV, because rGn-Fc (14/15) had broad cross reactivity compare with rGc-Fc (9/15). 0921 showed the different amino acids of M segment with 062904 and 062205, of which amino acids responsible for binding ability could be illustrated when monoclonal antibody was baited by panned from antibody library in the future. It may be shocking that the three donors (0921, 062904, and 062205) were fatal when infected with SFTSV and advanced age factors. It is necessary to analyze the relationship between genotype of SFTSV and mortality of subjects in the future.

Specific immunity plays an important role in viral infectious diseases. In a study by Jie Zhang et al., they found 1-year sustained cellular and humoral immunities in convalescents of COVID-19 (Zhang et al., 2021). In another study by Huang et al., they found that hospitalized patients with SFTS produced long-lasting neutralizing antibodies to SFTSV for 4 years (Huang et al., 2016). Along similar lines, Yoo et al. found the titers of neutralizing antibodies played an important role in protective immunity to SFTSV in survivors, and healthy residents who lived in endemic areas and who were positive for SFTSV IgG were higher than those in non-survivor patients (Yoo et al., 2021). It’s worth noting that antibodies from acute phase donors (X and Z) showed antibody dependent enhancement (ADE), which appeared the early infection. Surprisingly, donor X showed ADE phenomenon along with virus load (8.13E+02), then produced nAb when virus target did not determined (Cao et al., 2022). Based on these studies, we speculate single, or combination of different monoclonal nAb has the potential to be developed into a therapeutic agent.

NAbs play an effective role in emergency prevention and treatment. To explore the cross-neutralization of nAb of sera from SFTS patients, we examined sera from 25 convalescent patients. The results showed five nAbs (A2, A4, A5, L9, and L10) neutralized all six strains examined, which suggested the humoral immunity of SFTS patients produced cross-neutralizing antibodies to treat infections from SFTSV. Therefore, blood sources that produced cross nAbs could be used to construct antibody libraries to screen human monoclonal broad and potent nAbs for the treatment of SFTS in the future. This study thus provides a reliable preliminary basis for vaccine design.

## Conclusion

In summary, the epidemic of tick-borne SFTSV mainly occurred in eastern Asia. SFTS patients showed clinical symptoms of fever, thrombocytopenia, and leukocytopenia, along with hepatic, cardiac, and nerve system injury. C2 and C3 are the major epidemic strains in the Jiaodong area of Shandong Province, even so, genotypic diversity of SFTSV is needed a further survey limited to study population in Jiaodng area. There were cross nAbs in the sera of SFTS subjects who neutralized five genotype strains of SFTSV, which highlighted the important role of humoral immunity in combating SFTSV. Our data showed the possibility of screening human monoclonal broad and potent nAbs from the antibody library for the treatment of SFTS in the future. Moreover, detection of nAb and clades of SFTSV from sera of patients can aid clinicians in determining the serious trends of SFTS for timely treatment of patients.

## Data availability statement

The datasets presented in this study can be found in online repositories. The names of the repository/repositories and accession number(s) can be found in the article/Supplementary material.

## Ethics statement

The studies involving human participants were reviewed and approved by Binzhou Medical University and Yantai Qishan Hospital. The patients/participants provided their written informed consent to participate in this study.

## Author contributions

YQ designed the study, and wrote the initial draft of the manuscript, revising it critically for intellectual content. YL and XW collected the samples and communicated with patients, and the final approval of the version to be published. JX, FZ, JZ, and YB collected case data and conducted the experiments. WH analyzed data by statistical tool. YuW and BL supervised the study. YoW provided the experimental assistance. All authors contributed to the article and approved the submitted version.

## Funding

This work was supported by Natural Science Foundation of Shandong Province (ZR2020MH169).

## Conflict of interest

The authors declare that the research was conducted in the absence of any commercial or financial relationships that could be construed as a potential conflict of interest.

## Publisher’s note

All claims expressed in this article are solely those of the authors and do not necessarily represent those of their affiliated organizations, or those of the publisher, the editors and the reviewers. Any product that may be evaluated in this article, or claim that may be made by its manufacturer, is not guaranteed or endorsed by the publisher.
